# Supplementary material for: HIVprotI: an integrated web based platform for prediction and design of HIV proteins inhibitors
Source: J Cheminform. 2018 Mar 9;10:12. doi: 10.1186/s13321-018-0266-y (PMC5845081; doi:10.1186/s13321-018-0266-y)
Supplement: Supplementary file 2 — Additional file 2. Source code of HIVProtI web server. [file 13321_2018_266_MOESM2_ESM.zip › HIVprotI_Source-code/hivprotI/weka-3-6-6/doc/weka/classifiers/trees/REPTree.html]

REPTree


---


|  |  |  |
| --- | --- | --- |
| **Overview** \| **Package** \| **Class** \| **Tree** \| **Deprecated** \| **Index** \| **Help** \| **Weka's home** | |  |
| **PREV CLASS**   **NEXT CLASS** | **FRAMES**    **NO FRAMES**     **All Classes** |  |
| SUMMARY: NESTED \| FIELD \| CONSTR \| METHOD | DETAIL: FIELD \| CONSTR \| METHOD |  |


---


## weka.classifiers.trees Class REPTree

```
java.lang.Object
  weka.classifiers.Classifier
      weka.classifiers.trees.REPTree
```

**All Implemented Interfaces:**: java.io.Serializable, java.lang.Cloneable, Sourcable, AdditionalMeasureProducer, CapabilitiesHandler, Drawable, OptionHandler, RevisionHandler, WeightedInstancesHandler

---

``` public class REPTree extends Classifier implements OptionHandler, WeightedInstancesHandler, Drawable, AdditionalMeasureProducer, Sourcable ```

Fast decision tree learner. Builds a decision/regression tree using information gain/variance and prunes it using reduced-error pruning (with backfitting). Only sorts values for numeric attributes once. Missing values are dealt with by splitting the corresponding instances into pieces (i.e. as in C4.5).


Valid options are:

```
 -M <minimum number of instances>
  Set minimum number of instances per leaf (default 2).
```

```
 -V <minimum variance for split>
  Set minimum numeric class variance proportion
  of train variance for split (default 1e-3).
```

```
 -N <number of folds>
  Number of folds for reduced error pruning (default 3).
```

```
 -S <seed>
  Seed for random data shuffling (default 1).
```

```
 -P
  No pruning.
```

```
 -L
  Maximum tree depth (default -1, no maximum)
```

**Version:**
:   $Revision: 6952 $

**Author:**
:   Eibe Frank (eibe@cs.waikato.ac.nz)

**See Also:**: Serialized Form

---

**Field Summary**

**Fields inherited from interface weka.core.Drawable**

`BayesNet, Newick, NOT_DRAWABLE, TREE`


**Constructor Summary**

`REPTree()`


| **Method Summary** | |
| --- | --- |
| `void` | `buildClassifier(Instances data)`             Builds classifier. |
| `double[]` | `distributionForInstance(Instance instance)`             Computes class distribution of an instance using the tree. |
| `java.util.Enumeration` | `enumerateMeasures()`             Returns an enumeration of the additional measure names. |
| `Capabilities` | `getCapabilities()`             Returns default capabilities of the classifier. |
| `int` | `getMaxDepth()`             Get the value of MaxDepth. |
| `double` | `getMeasure(java.lang.String additionalMeasureName)`             Returns the value of the named measure. |
| `double` | `getMinNum()`             Get the value of MinNum. |
| `double` | `getMinVarianceProp()`             Get the value of MinVarianceProp. |
| `boolean` | `getNoPruning()`             Get the value of NoPruning. |
| `int` | `getNumFolds()`             Get the value of NumFolds. |
| `java.lang.String[]` | `getOptions()`             Gets options from this classifier. |
| `java.lang.String` | `getRevision()`             Returns the revision string. |
| `int` | `getSeed()`             Get the value of Seed. |
| `java.lang.String` | `globalInfo()`             Returns a string describing classifier |
| `java.lang.String` | `graph()`             Outputs the decision tree as a graph |
| `int` | `graphType()`             Returns the type of graph this classifier represents. |
| `java.util.Enumeration` | `listOptions()`             Lists the command-line options for this classifier. |
| `static void` | `main(java.lang.String[] argv)`             Main method for this class. |
| `java.lang.String` | `maxDepthTipText()`             Returns the tip text for this property |
| `java.lang.String` | `minNumTipText()`             Returns the tip text for this property |
| `java.lang.String` | `minVariancePropTipText()`             Returns the tip text for this property |
| `java.lang.String` | `noPruningTipText()`             Returns the tip text for this property |
| `java.lang.String` | `numFoldsTipText()`             Returns the tip text for this property |
| `int` | `numNodes()`             Computes size of the tree. |
| `java.lang.String` | `seedTipText()`             Returns the tip text for this property |
| `void` | `setMaxDepth(int newMaxDepth)`             Set the value of MaxDepth. |
| `void` | `setMinNum(double newMinNum)`             Set the value of MinNum. |
| `void` | `setMinVarianceProp(double newMinVarianceProp)`             Set the value of MinVarianceProp. |
| `void` | `setNoPruning(boolean newNoPruning)`             Set the value of NoPruning. |
| `void` | `setNumFolds(int newNumFolds)`             Set the value of NumFolds. |
| `void` | `setOptions(java.lang.String[] options)`             Parses a given list of options. |
| `void` | `setSeed(int newSeed)`             Set the value of Seed. |
| `java.lang.String` | `toSource(java.lang.String className)`             Returns the tree as if-then statements. |
| `java.lang.String` | `toString()`             Outputs the decision tree. |

**Methods inherited from class weka.classifiers.Classifier**

`classifyInstance, debugTipText, forName, getDebug, makeCopies, makeCopy, setDebug`

**Methods inherited from class java.lang.Object**

`equals, getClass, hashCode, notify, notifyAll, wait, wait, wait`

**Constructor Detail**

### REPTree

```
public REPTree()
```


**Method Detail**

### globalInfo

```
public java.lang.String globalInfo()
```

:   Returns a string describing classifier

    :   **Returns:**: a description suitable for displaying in the explorer/experimenter gui

---


### noPruningTipText

```
public java.lang.String noPruningTipText()
```

:   Returns the tip text for this property

    :   **Returns:**: tip text for this property suitable for displaying in the explorer/experimenter gui

---


### getNoPruning

```
public boolean getNoPruning()
```

:   Get the value of NoPruning.

    :   **Returns:**: Value of NoPruning.

---


### setNoPruning

```
public void setNoPruning(boolean newNoPruning)
```

:   Set the value of NoPruning.

    :   **Parameters:**: `newNoPruning` - Value to assign to NoPruning.

---


### minNumTipText

```
public java.lang.String minNumTipText()
```

:   Returns the tip text for this property

    :   **Returns:**: tip text for this property suitable for displaying in the explorer/experimenter gui

---


### getMinNum

```
public double getMinNum()
```

:   Get the value of MinNum.

    :   **Returns:**: Value of MinNum.

---


### setMinNum

```
public void setMinNum(double newMinNum)
```

:   Set the value of MinNum.

    :   **Parameters:**: `newMinNum` - Value to assign to MinNum.

---


### minVariancePropTipText

```
public java.lang.String minVariancePropTipText()
```

:   Returns the tip text for this property

    :   **Returns:**: tip text for this property suitable for displaying in the explorer/experimenter gui

---


### getMinVarianceProp

```
public double getMinVarianceProp()
```

:   Get the value of MinVarianceProp.

    :   **Returns:**: Value of MinVarianceProp.

---


### setMinVarianceProp

```
public void setMinVarianceProp(double newMinVarianceProp)
```

:   Set the value of MinVarianceProp.

    :   **Parameters:**: `newMinVarianceProp` - Value to assign to MinVarianceProp.

---


### seedTipText

```
public java.lang.String seedTipText()
```

:   Returns the tip text for this property

    :   **Returns:**: tip text for this property suitable for displaying in the explorer/experimenter gui

---


### getSeed

```
public int getSeed()
```

:   Get the value of Seed.

    :   **Returns:**: Value of Seed.

---


### setSeed

```
public void setSeed(int newSeed)
```

:   Set the value of Seed.

    :   **Parameters:**: `newSeed` - Value to assign to Seed.

---


### numFoldsTipText

```
public java.lang.String numFoldsTipText()
```

:   Returns the tip text for this property

    :   **Returns:**: tip text for this property suitable for displaying in the explorer/experimenter gui

---


### getNumFolds

```
public int getNumFolds()
```

:   Get the value of NumFolds.

    :   **Returns:**: Value of NumFolds.

---


### setNumFolds

```
public void setNumFolds(int newNumFolds)
```

:   Set the value of NumFolds.

    :   **Parameters:**: `newNumFolds` - Value to assign to NumFolds.

---


### maxDepthTipText

```
public java.lang.String maxDepthTipText()
```

:   Returns the tip text for this property

    :   **Returns:**: tip text for this property suitable for displaying in the explorer/experimenter gui

---


### getMaxDepth

```
public int getMaxDepth()
```

:   Get the value of MaxDepth.

    :   **Returns:**: Value of MaxDepth.

---


### setMaxDepth

```
public void setMaxDepth(int newMaxDepth)
```

:   Set the value of MaxDepth.

    :   **Parameters:**: `newMaxDepth` - Value to assign to MaxDepth.

---


### listOptions

```
public java.util.Enumeration listOptions()
```

:   Lists the command-line options for this classifier.

    :   **Specified by:**: `listOptions` in interface `OptionHandler` **Overrides:**: `listOptions` in class `Classifier`
    :   **Returns:**: an enumeration over all commandline options

---


### getOptions

```
public java.lang.String[] getOptions()
```

:   Gets options from this classifier.

    :   **Specified by:**: `getOptions` in interface `OptionHandler` **Overrides:**: `getOptions` in class `Classifier`
    :   **Returns:**: the options for the current setup

---


### setOptions

```
public void setOptions(java.lang.String[] options)
                throws java.lang.Exception
```

:   Parses a given list of options.


    Valid options are:

    ```
     -M <minimum number of instances>
      Set minimum number of instances per leaf (default 2).
    ```

    ```
     -V <minimum variance for split>
      Set minimum numeric class variance proportion
      of train variance for split (default 1e-3).
    ```

    ```
     -N <number of folds>
      Number of folds for reduced error pruning (default 3).
    ```

    ```
     -S <seed>
      Seed for random data shuffling (default 1).
    ```

    ```
     -P
      No pruning.
    ```

    ```
     -L
      Maximum tree depth (default -1, no maximum)
    ```

    :   **Specified by:**: `setOptions` in interface `OptionHandler` **Overrides:**: `setOptions` in class `Classifier`
    :   **Parameters:**: `options` - the list of options as an array of strings **Throws:**: `java.lang.Exception` - if an option is not supported

---


### numNodes

```
public int numNodes()
```

:   Computes size of the tree.

    :   **Returns:**: the number of nodes

---


### enumerateMeasures

```
public java.util.Enumeration enumerateMeasures()
```

:   Returns an enumeration of the additional measure names.

    :   **Specified by:**: `enumerateMeasures` in interface `AdditionalMeasureProducer`
    :   **Returns:**: an enumeration of the measure names

---


### getMeasure

```
public double getMeasure(java.lang.String additionalMeasureName)
```

:   Returns the value of the named measure.

    :   **Specified by:**: `getMeasure` in interface `AdditionalMeasureProducer`
    :   **Parameters:**: `additionalMeasureName` - the name of the measure to query for its value **Returns:**: the value of the named measure **Throws:**: `java.lang.IllegalArgumentException` - if the named measure is not supported

---


### getCapabilities

```
public Capabilities getCapabilities()
```

:   Returns default capabilities of the classifier.

    :   **Specified by:**: `getCapabilities` in interface `CapabilitiesHandler` **Overrides:**: `getCapabilities` in class `Classifier`
    :   **Returns:**: the capabilities of this classifier **See Also:**: `Capabilities`

---


### buildClassifier

```
public void buildClassifier(Instances data)
                     throws java.lang.Exception
```

:   Builds classifier.

    :   **Specified by:**: `buildClassifier` in class `Classifier`
    :   **Parameters:**: `data` - the data to train with **Throws:**: `java.lang.Exception` - if building fails

---


### distributionForInstance

```
public double[] distributionForInstance(Instance instance)
                                 throws java.lang.Exception
```

:   Computes class distribution of an instance using the tree.

    :   **Overrides:**: `distributionForInstance` in class `Classifier`
    :   **Parameters:**: `instance` - the instance to compute the distribution for **Returns:**: the computed class probabilities **Throws:**: `java.lang.Exception` - if computation fails

---


### toSource

```
public java.lang.String toSource(java.lang.String className)
                          throws java.lang.Exception
```

:   Returns the tree as if-then statements.

    :   **Specified by:**: `toSource` in interface `Sourcable`
    :   **Parameters:**: `className` - the name for the generated class **Returns:**: the tree as a Java if-then type statement **Throws:**: `java.lang.Exception` - if something goes wrong

---


### graphType

```
public int graphType()
```

:   Returns the type of graph this classifier
    represents.

    :   **Specified by:**: `graphType` in interface `Drawable`
    :   **Returns:**: Drawable.TREE

---


### graph

```
public java.lang.String graph()
                       throws java.lang.Exception
```

:   Outputs the decision tree as a graph

    :   **Specified by:**: `graph` in interface `Drawable`
    :   **Returns:**: the tree as a graph **Throws:**: `java.lang.Exception` - if generation fails

---


### toString

```
public java.lang.String toString()
```

:   Outputs the decision tree.

    :   **Overrides:**: `toString` in class `java.lang.Object`
    :   **Returns:**: a string representation of the classifier

---


### getRevision

```
public java.lang.String getRevision()
```

:   Returns the revision string.

    :   **Specified by:**: `getRevision` in interface `RevisionHandler` **Overrides:**: `getRevision` in class `Classifier`
    :   **Returns:**: the revision

---


### main

```
public static void main(java.lang.String[] argv)
```

:   Main method for this class.

    :   **Parameters:**: `argv` - the commandline options


---


|  |  |  |
| --- | --- | --- |
| **Overview** \| **Package** \| **Class** \| **Tree** \| **Deprecated** \| **Index** \| **Help** \| **Weka's home** | |  |
| **PREV CLASS**   **NEXT CLASS** | **FRAMES**    **NO FRAMES**     **All Classes** |  |
| SUMMARY: NESTED \| FIELD \| CONSTR \| METHOD | DETAIL: FIELD \| CONSTR \| METHOD |  |


---
